# Cold atmospheric plasma coupled with air abrasion in liquid medium for the treatment of peri-implantitis model grown with a complex human biofilm: an in vitro study

**DOI:** 10.1007/s00784-021-03949-x

**Published:** 2021-04-24

**Authors:** Wang Lai Hui, Vittoria Perrotti, Adriano Piattelli, Kostya (Ken) Ostrikov, Zhi Fang, Alessandro Quaranta

**Affiliations:** 1 Smile Specialists Suite, Newcastle, NSW Australia; 2 Formerly, School of Dentistry and Oral Health, Griffith University, Gold Coast, Queensland Australia; 3 Department of Medical, Oral and Biotechnological Sciences (DSMOB), University of Chieti-Pescara, Via dei vestini, 31, 66100 Chieti, Italy; 4 School of Chemistry and Physics, Queensland University of Technology, Brisbane, Queensland 4000 Australia; 5 College of Electrical Engineering and Control Science, Nanjing Tech University, Nanjing, 210009 China; 6 Sydney Dental Hospital, Sydney, NSW Australia

**Keywords:** Air abrasion, Biofilm, Decontamination, Dental implants, Plasma

## Abstract

**Objective:**

Treatment of implants with peri-implantitis is often unsuccessful due to residual microbial biofilm hindering re-osseointegration. The aim of this study was to treat biofilm-grown titanium (Ti) implants with different modalities involving air abrasion (AA) and cold atmospheric plasma (CAP) to compare the effectiveness in surface decontamination and the alteration/preservation of surface topography.

**Materials and methods:**

Saliva collected from a peri-implantitis patient was used to in vitro develop human biofilm over 35 implants with moderately rough surface. The implants were then mounted onto standardized acrylic blocks simulating peri-implantitis defects and treated with AA (erythritol powder), CAP in a liquid medium, or a combination (COM) of both modalities. The remaining biofilm was measured by crystal violet (CV). Surface features and roughness before and after treatment were assessed by scanning electron microscope (SEM). The data were statistically analyzed using Kruskal-Wallis followed by Tukey’s multiple comparison test.

**Results:**

In the present peri-implantitis model, the human complex biofilm growth was successful as indicated by the statistical significance between the negative and positive controls. All the treatment groups resulted in a remarkable implant surface decontamination, with values very close to the negative control for AA and COM. Indeed, statistically significant differences in the comparison between the positive control vs. all the treatment groups were found. SEM analysis showed no post-treatment alterations on the implant surface in all the groups.

**Conclusions:**

Decontamination with AA delivering erythritol with or without CAP in liquid medium demonstrated compelling efficacy in the removal of biofilm from implants. All the tested treatments did not cause qualitative alterations to the Ti surface features. No specific effects of the CAP were observed, although further studies are necessary to assess its potential as monotherapy with different settings or in combination with other decontamination procedures.

**Clinical relevance:**

CAP is a promising option in the treatment of peri-implantitis because it has potential to improve the elimination of bacterial plaque from implant surfaces, in inaccessible pockets or during open-flap debridement, and should stimulate the process of the re-osseointegration of affected dental implants by not altering surface features and roughness.

## Introduction

Biofilm-induced implant disease, peri-implantitis, has been reported to occur in 1–47% of all implants placed globally [1]. Despite the high prevalence, currently scientists are still in perusal for a universally accepted and reliable treatment modality. Peri-implantitis is an inflammatory reaction of the host to bacteria and presents clinically with pathological bone loss around dental implants which often results in saucer-shaped bony defects [2]. The ultimate outcome of untreated or failed treatment of peri-implantitis is the loss of the implant. While regular professional and home care can maintain healthy peri-implant tissues, once a mature biofilm has established itself on the subgingival implant surface, it becomes a big challenge for management [3]. This is because the implant surface is both macroscopically and microscopically rough, which makes it notoriously difficult to clean. Indeed, early dental implants used to have a simple threaded screw forms and plain, machined surfaces which were unmodified after milling, while almost all modern dental implants are designed to have rough surfaces with features that increase the surface area and energy. The rationale is to enhance the adhesion of blood, matrix proteins, and human cells [4]. Altering the surface of a titanium (Ti) implant to increase its roughness does not compromise its biocompatibility but enhances the total area available for host cells attachment, hence integration with bone osseointegration [5]. Unfortunately, these altered implant surfaces with complex topographies do not differentiate between prokaryotes and eukaryotes. In other words, both bacterial and host cells can attach to the implants simultaneously. This effect promotes biofilm formation, the leading etiology of peri-implantitis.

Despite many trials on peri-implantitis treatments, multiple systematic reviews have yielded the same conclusion that based on the current evidence, no particular treatment can be established as a gold standard approach for the treatment of peri-implantitis [6–9]. Since the treatment of peri-implantitis aims to achieve both decontamination and re-osseointegration, it is necessary to remove not only all viable bacteria but all traces of bacterial products such as endotoxins, in order to maximize the likelihood of success. Traditional periodontal treatments using scaling instruments to remove biofilms originally designed for debriding the roots of natural teeth cannot be applied in the same manner to threaded implant surfaces. The implant surface is far more protected and inaccessible to conventional professional instruments [10]; this lack of cleaning efficacy is worse in non-surgical approach. This is particularly true when the peri-implant lesions are compounded by unfavorable defect configurations, for example, the typical deep and narrow bony defects; these findings necessitated exploration of surgical options [11]. As shown in various systematic reviews [12, 13], while non-surgical treatment has been shown to be effective in resolving peri-implant mucositis, it does not reliably resolve peri-implantitis. Peri-implantitis is best managed surgically.

Nevertheless, whether it is conservative, resective, or regenerative treatments, surgical approaches would only be successful if they are executed in conjunction with effective implant surface decontamination [11]. Access to the implant surface may be improved by surgical means, but it is still far from ideal; most of the techniques proposed in the scientific literature are limited by lack of adequate access. In vitro peri-implantitis models stained with ink and tested for air abrasion (AA) with several powder particles reported to have the superior surfaces of the implant threads more readily cleaned, whereas the inferior surfaces are often observed to retain residual dye [10, 14]. Dental laser applications in treating peri-implantitis are also exploring side-firing tips to more efficiently access the under-surface of the implant threads [15, 16]. AA has demonstrated its superior biofilm decontaminating power on flat titanium discs, but its efficacy in peri-implantitis model is yet to be tested [17].

Cold atmospheric plasma (CAP) is an innovative, yet early-stage, treatment modality in the oral implantology field. In a recent review [18] based on 23 studies investigating the effects of CAP on biocompatibility, surface improvement, and cleaning efficacy of implant surfaces, it was suggested that CAP is a promising option for the treatment of peri-implantitis [19, 20], although further evidence is necessary to draw final conclusions. The hypothesized benefits in incorporating CAP for peri-implantitis treatment is that it can act via a liquid medium. The liquid environment, in which the cells are normally located, acts as an interface between plasma and living matter. CAP can activate a liquid interface with reactive species which then act as a carrier agent to deliver the antibacterial effects onto the underlying target surface, as simulated by dental implants contaminated by biofilm treatment in a combination of saliva, gingival crevicular fluid, and even blood. Tresp et al. (2013) [21] showed superoxide anion radicals (O_2_^•-^), as well as hydroxyl radicals (^•^OH) were generated in plasma-treated phosphate-buffered saline solution. Earlier in 2010, Liu et al. [22] successfully inactivated *Staphylococcus aureus* suspended in a liquid using a direct-current CAP microjet. This finding has prompted the potential use of CAP in treating peri-implantitis despite the lack of direct contact between the plasma jet and the implant surface.

The aim of this study was to treat biofilm-grown Ti implants in a peri-implantitis model with different modalities involving AA and CAP in order to compare their efficacy in surface decontamination and the effects of those decontamination on Ti surface features.

## Materials and methods

### Titanium implants

A total of 35 grade 4 pure Ti dental implants (4.1 × 10mm) with a moderately rough surface blasted with alumina particles (Southern Implants®, Irene, South Africa) were adopted for the present study.

### Study design

The protocol of the present study was approved by the Griffith University Human Research Ethics Committee (GU Ref No: 2018/35).

**Fig. 1:**
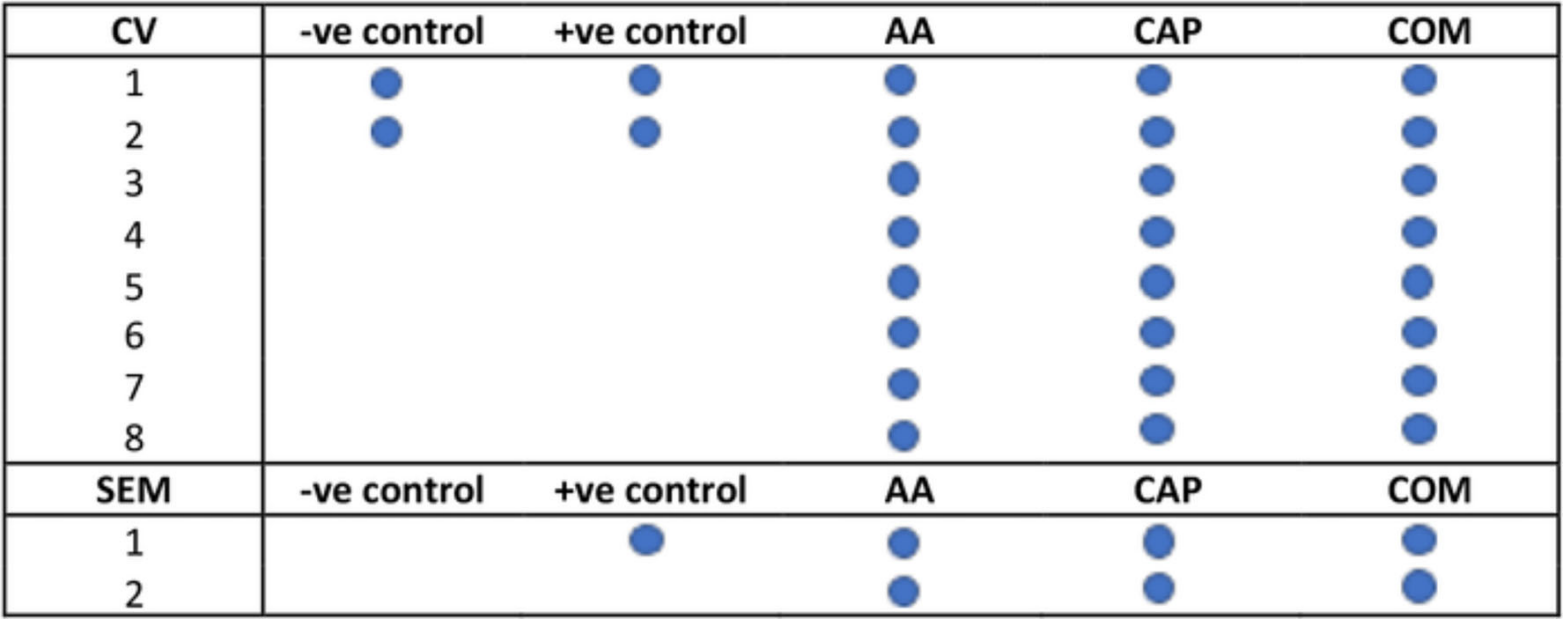
Schematic drawing of the study design

The 35 Ti implants are subdivided into 5 treatment groups (Fig. 1):

−ve control group: non-contaminated and treated by AA and CAP (#2)
- +ve control group: contaminated and untreated (# 3)
- AA: contaminated and treated the by AA (#10)
- CAP: contaminated and treated by CAP (#10)
- COM: contaminated and treated by the combined AA and CAP treatment (#10)

### Biofilm growth

A systemic healthy volunteer with no evidence of active caries, no salivary gland disease, but affected by peri-implantitis was selected to donate his saliva. The subject was asked to refrain from practicing oral hygiene routine for 12 h before saliva collection in the morning. The subject chewed on paraffin wax until 5mL of saliva were collected.

To generate biofilm, Ti implants were incubated in 1mL of Brain Heart infusion (BHI) media (OXOID CM1135, 37gr/L) supplemented with 5% defibrinated sheep blood [23] and 10% human saliva from the donor [24, 25] in sterile 24-well plates (Costa 3524) for 96 h under anaerobic conditions at 37°C, 80rpm to allow bacterial growth. For negative controls, implants were incubated at the same conditions with 1mL of BHI supplemented with 5% defibrinated sheep blood and 10% PBS solution, but without human saliva.

### Peri-implantitis model

**Fig. 2:**
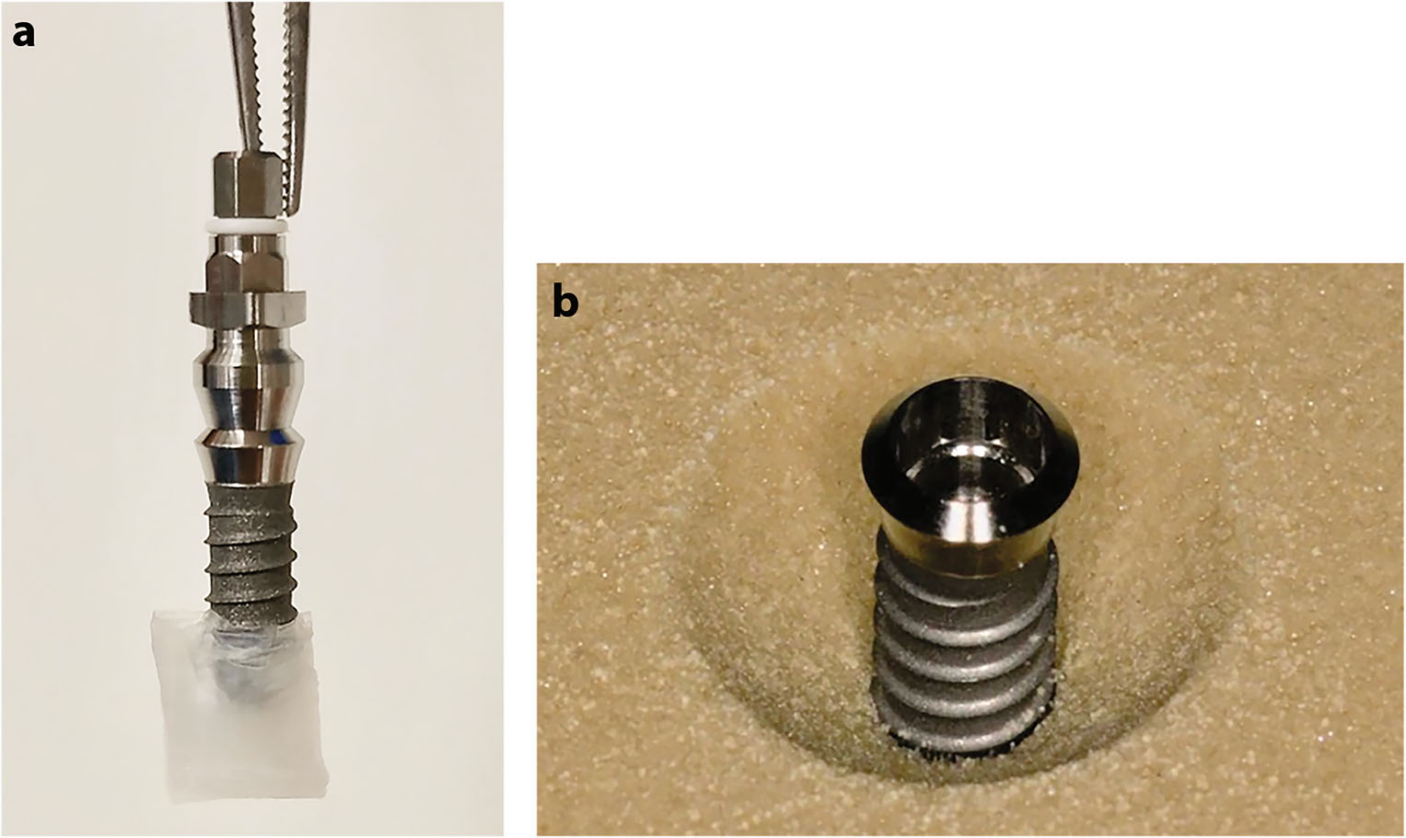
**a** Parafilm wax coating of implant’s apical 4mm. **b** Peri-implantitis model of Sawbone defect

Prior to biofilm growth, the apical 4mm of the 35 implants was coated with parafilm wax (Fig. 2a). As the biofilm matured on the implant surface, the parafilm wax was removed; hence, only the coronal 6mm of the implant surface and the tissue level machined collar were contaminated with biofilm growth. Each implant was mounted in an acrylic resin block (Sawbones, Vashon Island, WA, USA) prepared with 6-mm-deep defects with a circumferential saucer-shaped opening at 60°C (Fig. 2b) to simulate the physical environment of a peri-implantitis lesion [14]. These defects were in the same morphology as the Class Ie defects described by Schwarz et al. [26]. When the implants were inserted into the pre-fabricated defects, three full threads were exposed in the coronal region.

### Instrumentation modalities

#### Air abrasion

**Fig. 3:**
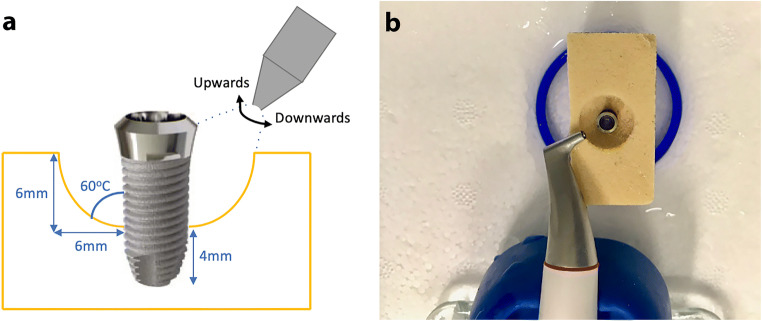
**a** Schematic drawing of air abrasion treatment. **b** Setup of PI model and air abrasion treatment

Implants were treated on 4 surfaces with 90° rotation per surface in accordance with the acrylic resin block. An AA device (Airflow® Prophylaxis Master, EMS, Nyon, Switzerland) at a static pressure 7 bar (101.5 psi) using erythritol powder (particle size 14 μm) was used to treat the implants for 20 s in each surface, 10 s upwards and 10 s downwards motion with the nozzle head angulated towards the mid-point of the treated implant surface, at a distance of 10 mm and 60mL water/min. A standard handpiece (Airflow®, EL-308/C, EMS Nyon, Switzerland) was mounted with a holder so as to keep the nozzle at a static position to each Ti implants treated. The holder allows rotation of the handpiece along its own axis so as to ensure that ejected water, powder, and air are projected along the implant surface (Fig. 3a–b). All treatments were performed by the same experienced operator, a senior periodontics specialist in training (W.H.L.).

#### Cold atmospheric plasma

**Fig. 4:**
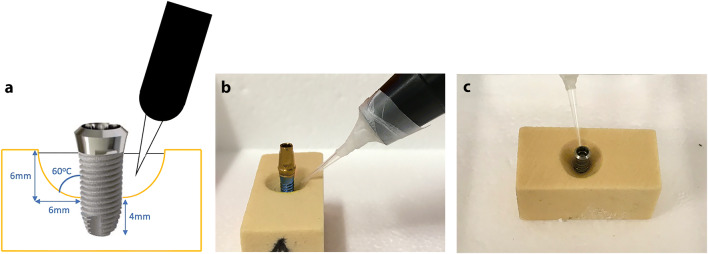
**a** Schematic drawing of CAP treatment. **b** Setup of CAP treatment (NB: implant not representative). **c** Setup of CAP treatment

Plasma treatment was performed with an experimental spark plasma pen jet previously described in detail in Hui et al. [27]. The pen jet was mounted on a standardized holder keeping a distance of 5 mm from the plasma nozzle tip (end of discharge capillary) to the Ti implants surface. The plasma pulses were delivered with the repetition frequency of 1.4 Hz. A high RF voltage (10 kV) was coupled to the needle electrode. The temperature was maintained very close to the room temperature (RT), with the deviation not exceeding 2°C at the tip of the plasma jet. All roughened Ti implants surfaces were submerged in PBS solution to ensure that the liquid medium smoothly covers all the imperfections (e.g., rough surface features) on the sample surface (Fig. 4a–c).

### Biofilm quantification

Eight implants belonging to groups AA, CAP, and COM and 2 implants for negative and positive controls were assessed by crystal violet (CV) assay [28] to evaluate the effect of different treatment modalities on biofilm removal; experiments were repeated in triplicates. Implants were first washed twice with 500 μL of sterile distilled water. The biofilm was then stained with 500 μL of 0.1% CV for 30 min at room temperature in a 24-well plate. The implants were than washed three times with 1 mL of sterile distilled water and removed maximum water possible by tapping onto paper towel. The dye bound to adherent biofilm was then solubilized using 500 μL of ethanol to acetone ratio of 80%:20%, and the optical density (OD) of the solubilized dye was measured at 595nm with a plate reader (POLARstar Omega plate reader, BMG Labtech, Adelaide, Australia). Ethanol to acetone ratio was used as the blank to subtract any background reading. The percentage reduction in biofilm caused by the treatment was calculated as follows:

$$ \left(\left({\mathsf{OD}}_{+\mathsf{ve}\ \mathsf{control}}\hbox{--} {\mathsf{OD}}_{\mathsf{treatment}}\right)/{\mathsf{OD}}_{+\mathsf{ve}\ \mathsf{control}}\right)\times \mathsf{100}\%. $$

### Measurement of surface topography

Pristine implants (uncontaminated and untreated) were evaluated by scanning electron microscope (SEM, Thermal Field Emission SEM, JEOL JSM-7100, Tokyo, Japan) to provide a baseline surface assessment and description prior to group allocation. The samples were rinsed with 0.1 M of phosphate-buffered solution (PBS) at pH 7.1 and fixed overnight with a 4% PBS–paraformaldehyde solution at 4 °C. Samples were further washed with PBS buffer and dehydrated using an ascending alcohol series before mounting onto aluminum stubs and gold sputtering in an Emitech K550 (Emitech Ltd., Ashford, Kent, UK).

A SEM with 10 kV and 3.3 A was used to capture images of the implants surface before and after the treatments. These images were used to examine any alteration on the Ti surface and were captured at 3, 6, 9, and 12 o’clock positions, at a distance of 300 mm from the center of the implant. Two magnifications of micrographs were used: ×270 and ×2700.

### Statistical analysis

Statistical analyses were carried out using a specific software (GraphPad, CA, USA). The sample size was obtained assuming the standard deviation of 0.024 of the implant surface decontamination as the primary outcome [29]. It was calculated that 6 implants would be required in each group to have a fixed power of 80 % with an alpha risk of 5 % for the main variable. Data distribution was checked for normality by Shapiro-Wilk test. Kruskal-Wallis followed by Tukey’s multiple comparisons test was used to compare the differences among groups. Data were presented as means ± standard deviations (SDs) at each time (pre- and post-treatment) for every experimental group. Statistically significant differences were set to *p*< 0.05.

## Results

### Biofilm quantification

The amount of biofilm present on the implants in the different groups was measured through CV staining and then expressed by means ± standard deviation values of the OD_595 nm_, which reflects the CV’s absorbance (Table 1).

**Table 1:** The amount of residual biofilm following decontamination treatments, measured through crystal violet staining and expressed by means ± standard deviation values of the optical density measurements.

-ve control	+ve control	AA	CAP	COM
0.0215	0.136 ± 0.002	0.030 ± 0.003	0.078 ± 0.007	0.029 ± 0.004

Data are presented as mean ± standard deviation (SD)

-ve control, non contaminated and treated by air abrasion and cold atmospheric plasma; +ve control, contaminated and untreated; AA, air abrasive; CAP, cold atmospheric plasma; COM, air abrasive + cold atmospheric plasma

The human complex biofilm growth was successful as indicated by the statistical significance between the negative and positive controls. All the treatment groups (AA, CAP, and COM) resulted in a remarkable implant surface decontamination; statistically significant differences in the comparison between positive control vs. all the treatment groups were found. Besides, the groups AA and COM showed values of residual biofilm very close to the negative control with no statistical significance found in the comparison between negative control vs. AA (*p*=0.1951) and vs. COM (*p*=0.3013) as well as for AA vs. COM (*p*=0.9930) (Table 2).

**Table 2:** Results of Tukey’s multiple comparison test for biofilm quantification

	Mean rank diff.	*p* value
-ve control vs. +ve control	−0.1145	**** <0.001
-ve control vs. AA	−0.008500	0.1951
-ve control vs. CAP	−0.05650	**** <0.001
-ve control vs. COM	−0.007500	0.3013
+ve control vs. AA	0.1060	**** <0.001
+ve control vs. CAP	0.05800	**** <0.001
+ve control vs. COM	0.1070	**** <0.001
AA vs. CAP	−0.04800	**** <0.001
AA vs. COM	0.001000	0.9930
CAP vs. COM	0.04900	**** <0.001

-ve control, non contaminated and treated by air abrasion and cold atmospheric plasma; +ve control, contaminated and untreated; AA, air abrasive; CAP, cold atmospheric plasma; COM, air abrasive + cold atmospheric plasma

*p* values were based on Tukey’s multiple comparison test

**Fig. 5:**
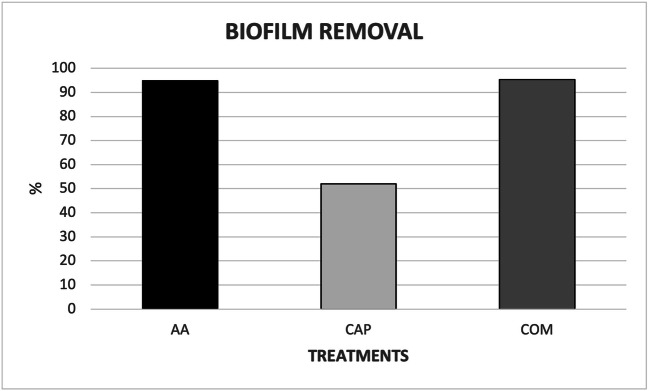
Percentage of biofilm removal after the decontamination of Ti implants with a moderately rough surface by means of air-abrasive unit (AA) delivering erythritol, cold atmospheric plasma (CAP), and combined AA and CAP (COM) treatments

The measured percentage of biofilm removal revealed an effective decontamination following all the treatments and almost complete biofilm removal after both AA application (94.87%) and when CAP was used in combination with AA in the COM treatment (95.32%). CAP alone showed 52.10% of biofilm removal (Fig. 5).

### Surface topography

**Fig. 6:**
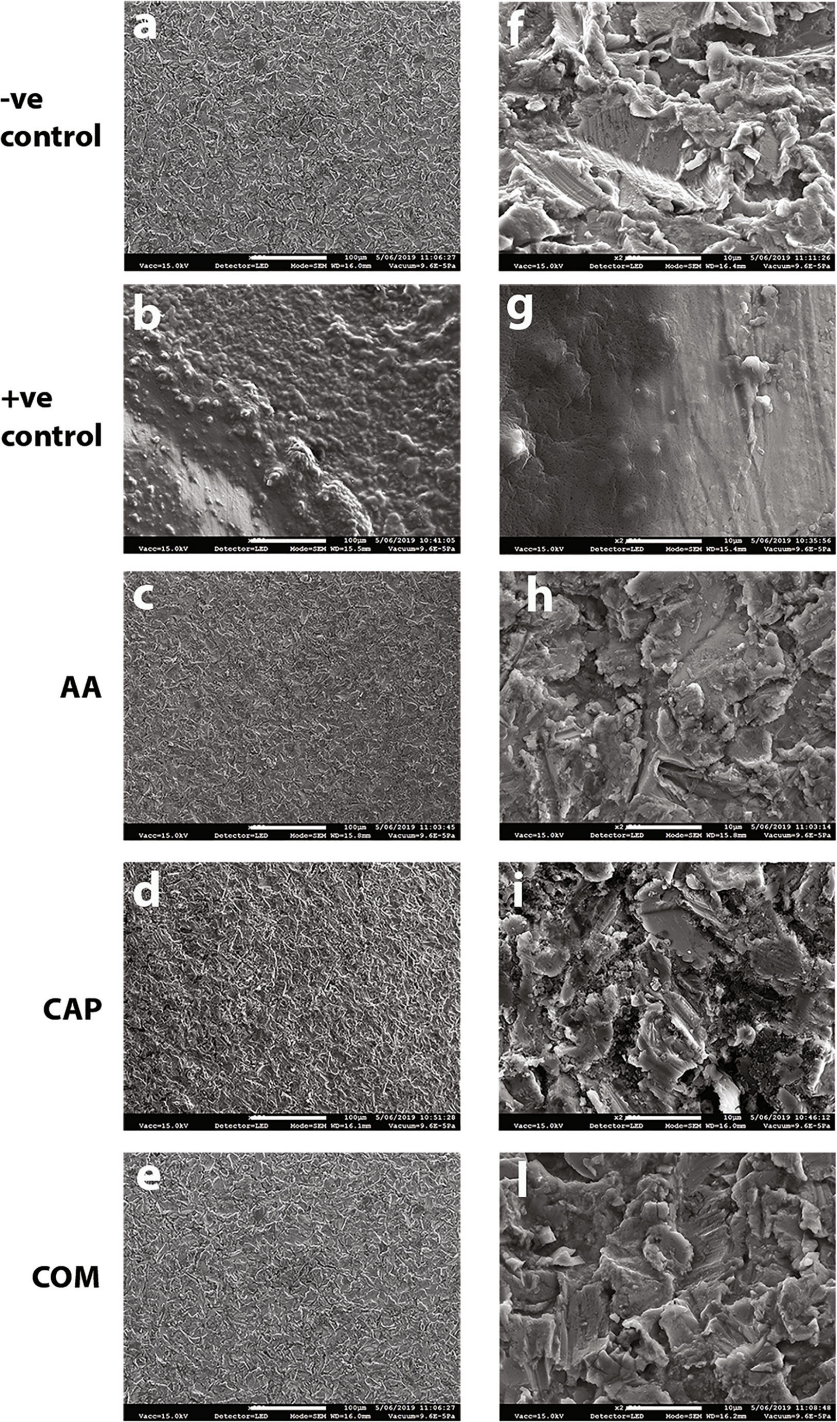
Scanning electron microscope (SEM) images of the titanium implants after the decontamination with air-abrasive unit (AA), cold atmospheric plasma (CAP), combined AA and CAP (COM) treatments; negative (-ve) control, non-contaminated and treated by AA and CAP; positive (+ve) control group, contaminated and untreated discs. Scale bars are 100 (**a**–**e**) and 10 (**f**–**l**) micrometers, magnification: **×**270 (**a**–**e**) and ×2700 (**f**–**l**).

SEM analysis at different magnifications outlined no post-treatment alterations on the implants, such as crater-like defects or scratches, indicating that none of the applied treatments led to surface feature changes. In addition, the post-treatment SEM images revealed the significant removal of biofilm with the AA and COM treatments. In the +ve control at both low- and high-power magnification, presumable remnants of biofilm obscured the original implant surface (Fig. 6a–l).

## Discussion

The ideal treatment for peri-implantitis should aim at the removal of the whole biofilm, the achievement of a sterile implant surface, and the maintenance of the implant surface topography; this in turn enhances host cell adhesion and re-osseointegration. The search for an efficient and safe approach for Ti surface decontamination has resulted in an effective treatment modality which is the AA treatment. Although there is no robust clinical evidence suggesting which decontamination modality is the most effective for treating peri-implantitis, a recent review [30] concluded that AA has a clinical efficacy which is superior or equal to all other decontamination methods (i.e.: Er:YAG laser, metal instruments and ultrasonic devices, plastic curettes, and rubber cups). Regarding the delivered powder, the best results were obtained using erythritol/chlorhexidine in terms of preventing regrowth of oral biofilm [30]. Based on the evidence mentioned above in the present study, it was decided to investigate AA delivering erythritol and complement the treatment with CAP. Indeed, CAP should be seen not only as an alternative but also as an adjunct to investigate synergistic treatment modalities. Very few studies have been published in the literature on the effects of a combined treatment of CAP with other devices. In a recent review [18], only 17 in vitro studies and 5 in vivo on animal models investigating the effects of CAP on biocompatibility, surface improvement, and cleaning efficacy were found in the literature. Among these studies, only Matthes et al. [31] assessed the effects of a combined biofilm removal with an optimized AA with erythritol powder and an argon CAP on osteoblast-like cells spreading. They concluded that CAP alone did not render the surface conducive for cells and that the combination was not superior to AA alone. These results were confirmed by a very recent study by our group [27] where a relevant decontamination efficacy of both machined and moderately rough surfaces following AA and COM treatments was found. In detail, treatment with AA (99.92% and 93.96) and COM (95.94% and 88.55%) resulted in higher decontamination compared to CAP 80.9% and 42.63%; however, no specific effect of the CAP in the combined treatment was observed. These results are in line with the ones achieved in the present study where AA showed 94.87% of biofilm removal, the combined treatment only a slight additional effect (95.32%), and CAP alone 52.10%, although it was used in a liquid environment. Plasma-liquid interactions have gained tremendous attention in the last few years; the transfer of reactivity from the gas to the liquid phase has been highlighted as being of prime importance for biological effects. Indeed, the depth of penetration of CAP can be limited to 60 μm, while CAP-activated liquid medium can act longer and deeper [32]. However, there are no indications in the literature about the exact “therapeutic dose”; the plasma gaseous products—and therefore the species generated in liquids—strongly depend on the discharge regime, its deposited power, and gas flow conditions. The gaseous products then determine the chemical properties of the CAP-activated liquid medium and the dominant aqueous reactive oxygen and nitrogen species (RONS). It was out of the scope of the present article to quantify the RONS produced by the investigated CAP device in our experimental conditions. Hence, an excessive dilution might be the cause of the slight effect of CAP alone and in combination.

The limitations of the present study are related to the restricted thickness of biofilms to several micrometers and the variation of the thickness due to its inhomogeneous growth. Another limitation concerns the plasma device itself. Indeed, it is an experimental device that was constructed to test different plasma parameters in vitro and to understand the principles of cold plasma generation. Therefore, further process optimization should be implemented to use it as a medical device, and its biocompatibility to oral mucosa should be investigated [33, 34]. Our results should be just understood mostly as a proof of principle. Nevertheless, we can state that plasma is an effective antimicrobial agent and further engineering, and optimization of the plasma sources is a viable way towards clinical translation.

## Conclusions

Decontamination with AA delivering erythritol with or without CAP is highly effective in biofilm removal from titanium surfaces in our peri-implantitis model. All the tested treatments resulted in minimal titanium surface alterations that do not compromise the original surface features. Further studies are needed to confirm the decontamination effect of CAP as single treatment modality and its eventual additive or synergic effect when CAP is used in liquid medium combined with AA.
